# A journey to Semantic Web query federation in the life sciences

**DOI:** 10.1186/1471-2105-10-S10-S10

**Published:** 2009-10-01

**Authors:** Kei-Hoi Cheung, H Robert Frost, M Scott Marshall, Eric Prud'hommeaux, Matthias Samwald, Jun Zhao, Adrian Paschke

**Affiliations:** 1 Center for Medical Informatics, Yale University School of Medicine, New Haven, CT 06511 USA; 2 VectorC, LLC, Hanover, NH 03755 USA; 3 Informatics Institute, University of Amsterdam, The Netherlands; 4 World Wide Web Consortium, Massachusetts Institute of Technology, Massachusetts, MA 02139 USA; 5 Digital Enterprise Research Institute, National University of Ireland Galway, IDA Business Park, Lower Dangan, Galway Ireland; 6 Konrad Lorenz Institute for Evolution and Cognition Research, Altenberg, Austria; 7 Department of Zoology, University of Oxford, Oxford, OX1 3PS UK; 8 Freie Universität Berlin, Germany

**Keywords:** Resource Description Framework, SPARQL Query, AIDA, Uniform Resource Identifier, Link Open Data

## Abstract

**Background:**

As interest in adopting the Semantic Web in the biomedical domain continues to grow, Semantic Web technology has been evolving and maturing. A variety of technological approaches including triplestore technologies, SPARQL endpoints, Linked Data, and Vocabulary of Interlinked Datasets have emerged in recent years. In addition to the data warehouse construction, these technological approaches can be used to support dynamic query federation. As a community effort, the BioRDF task force, within the Semantic Web for Health Care and Life Sciences Interest Group, is exploring how these emerging approaches can be utilized to execute distributed queries across different neuroscience data sources.

**Methods and results:**

We have created two health care and life science knowledge bases. We have explored a variety of Semantic Web approaches to describe, map, and dynamically query multiple datasets. We have demonstrated several federation approaches that integrate diverse types of information about neurons and receptors that play an important role in basic, clinical, and translational neuroscience research. Particularly, we have created a prototype receptor explorer which uses OWL mappings to provide an integrated list of receptors and executes individual queries against different SPARQL endpoints. We have also employed the AIDA Toolkit, which is directed at groups of knowledge workers who cooperatively search, annotate, interpret, and enrich large collections of heterogeneous documents from diverse locations. We have explored a tool called "FeDeRate", which enables a global SPARQL query to be decomposed into subqueries against the remote databases offering either SPARQL or SQL query interfaces. Finally, we have explored how to use the vocabulary of interlinked Datasets (voiD) to create metadata for describing datasets exposed as Linked Data URIs or SPARQL endpoints.

**Conclusion:**

We have demonstrated the use of a set of novel and state-of-the-art Semantic Web technologies in support of a neuroscience query federation scenario. We have identified both the strengths and weaknesses of these technologies. While Semantic Web offers a global data model including the use of Uniform Resource Identifiers (URI's), the proliferation of semantically-equivalent URI's hinders large scale data integration. Our work helps direct research and tool development, which will be of benefit to this community.

## Background

As the number, size, and complexity of life science databases continue to grow, data integration remains a prominent problem in the life sciences. These disparate databases feature diverse types of data including sequences, genes, proteins, pathways, and drugs produced by different kinds of experiments, including those that involve high-throughput technologies such as DNA microarray, mass spectrometry, and next generation sequencing. The challenges involved in integrating such data include inconsistency in naming, diversity of data models, and heterogeneous data formats. The benefits of integrating these disparate sources of data include discovery of new associations/relationships between the data and validation of existing hypotheses.

Numerous life science databases can be accessed publicly via the Web. The data retrieved from different databases are displayed using the HyperText Markup Language (HTML) and rendered by Web browsers (e.g., Internet Explorer and Firefox). Hypertext links are used to connect data items between different Web pages. Data integration using hypertext links, however, is burdensome to the user [1]. HTML works well to expose the results of scripted (canned) queries but does not expose the database structure to data users who would wish to construct their own queries. To automate integration of data in HTML format, we need to rely on methods such as screen scraping to extract the data from the HTML documents and integrate the extracted data by custom scripts. This approach is vulnerable to changes in display and location of Web pages. Such changes, together with changes in database structure, significantly increase the code complexity of data integration. To address this problem, approaches have been developed to facilitate data integration on a larger scale. Some representative approaches include EBI SRS [2], Atlas [3], DiscoveryLink [4], Biokleisli [5], Biozon [6], etc. In general, these approaches fall into two categories: data warehouse and federated database. The data warehouse approach relies on data translation in which data from different databases are re-expressed in a common data model on a central repository. The federated approach features query translation in which data are kept in their local databases and a global query can be translated into a set of local database subqueries whose results are unified and presented to the user. There are pros and cons for each approach. Data warehouses typically wrestle with the concurrency issue (keeping the data up-to-date with respect to a data source). Each time a member database is changed, the data translation code will need to be modified and/or re-executed, depending on the nature of the change. On the other hand, data warehouse query performance is good because queries are run locally. In the federated approach, data concurrency is not an issue, but query speed may be slow, especially when large amounts of data are transferred over the network.

The Semantic Web [7] transforms the Web into a global database or knowledge base by providing: i) globally unique names through the Uniform Resource Identifiers (URI's), ii) standard languages including the Resource Description Framework (RDF), RDF Schema (RDFS), and the Web Ontology Language (OWL) for modeling data and creating ontologies, and iii) a standard query language – SPARQL [8]. Enabling technologies such as ontology editors (e.g., Protégé), OWL reasoners (e.g., Pellet and FaCT++) and triplestores with SPARQL endpoints (e.g., Virtuoso, AllegroGraph and Sesame) help make the Semantic Web vision a reality. While these core and enabling technologies are maturing, there are new technological developments that can help push the Semantic Web to a new level of data interoperability. For example, Linked Data [9] is a method of exposing, sharing, and connecting data via dereferenceable HTTP URI's on the Semantic Web. A dereferenceable HTTP URI serves as both an identifier and a locator. The key idea is that useful information should be provided to data consumers when its URI is dereferenced. Using the Linked Data approach, not only do data providers make their data available in the form of RDF graphs, but data linkers can also create new RDF graphs that consist of links between independently developed RDF graphs provided by different sources. Examples of Linked Data (e.g., DBpedia [10, 11]) are listed on Linking Open Data (LOD) [9]. Vocabulary of Interlinked Datasets (voiD) [12] is an emerging standard for using a RDF based schema to describe linked datasets accessible through dereferenceable HTTP URI's or SPARQL endpoints. It provides a common vocabulary that can be used to describe datasets to assist the discovery of relevant linked data; or be associated with each dereferenceable data URI, enabling search engines or query mediators to follow the links in the web of data.

The BioRDF task force is one of the several task forces in the Semantic Web for Health Care and Life Sciences Interest Group (HCLS IG) [13] chartered by the World Wide Web Consortium (W3C). HCLS IG develops, advocates for, and supports the use of Semantic Web technologies for biological science, translational medicine and health care. The different task forces [14] involve participants from different organizations in both the industry and academia, who work together to solve particular sets of problems using Semantic Web technologies. As a consequence of such community support, we have witnessed growth in the use and demonstration of Semantic Web technologies in life science data integration. To date, most of the Semantic Web data integration approaches in life sciences use the data warehouse method. As pointed out in [15], there is a need to invest more efforts in exploring how to use the Semantic Web to implement the federation approach. This paper describes a journey to realizing this implementation in the context of neuroscience data federation. The federation involves SPARQL endpoints, linked datasets, and other formats including spreadsheets and semantic tags. This work represents a community effort carried out by the BioRDF task force [16] within the charter of the HCLS IG.

### Neuroscience use case

In the previous charter of the HCLS IG, the BioRDF task force focused its effort on data conversion and integration in the neuroscience context. Two main projects were derived from this effort. One of them was the creation of a central knowledge base (HCLS KB) that housed a variety of biomedical data including neuroscience data that were converted into RDF/OWL. The other project involved conversion of several SenseLab databases (Oracle) including NeuronDB, ModelDB, and BrainPharm into OWL ontologies. Both projects were described in W3C, which were made available to the public. In addition, a demo at the WWW2007 conference in Banff, Alberta, Canada showed queries that integrated multiple datasets stored in the HCLS KB to answer specific neuroscience research questions.

**Figure 1:**
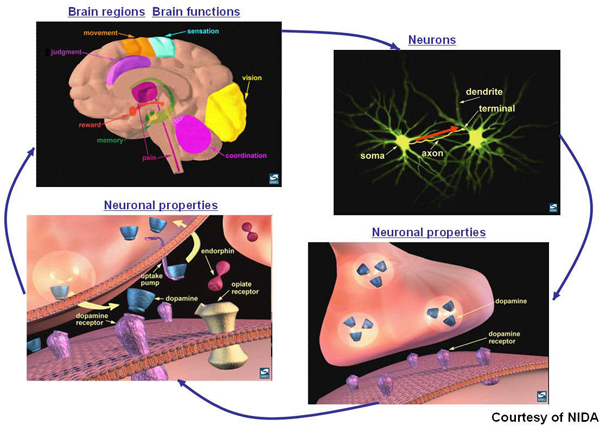
**Different types of brain data at different levels: anatomical level (top left image), neuronal level (top right image), and synaptic level (bottom two images)**.

In the renewed charter, the BioRDF task force leverages the previous effort to expand the neuroscience use case. Figure 1 depicts different categories of brain data across multiple levels including the anatomical level (top left image), neuronal level (top right image), and synaptic level (the bottom two images). At the anatomical level, the brain is divided into different regions. Different brain regions are responsible for different behaviors/functions. For example, the Hippocampus plays a major role in memory formation, while the Cerebellum is a brain region that is important for movement. At the neuronal level, neurons (special types of nerve cells) and their connections are of concern. A neuron has different parts: dendrites (receiving signals from other neurons), axons (transmitting signals to other neurons), and soma (directing activities of the neuron). Although most neurons contain all of the three parts, there is a wide range of diversity in the shapes and sizes of neurons as well as their axons and dendrites. The red arrow represents an action potential (electrical impulse) that travels from the soma to the end of the axon (terminal). This electrical impulse triggers the release of molecules called "neurotransmitters" (e.g., dopamine) from the axon terminal into the synapse (a tiny space between the axon terminal and dendrite of two connecting neurons). Before entering the synaptic space, neurotransmitters are contained in vesicles. The electrical impulse pushes these vesicles towards the cell membranes of the axon terminal. When the vesicles are in contact with the cell membranes, they break and neurotransmitters diffuse across the membrane into the synapse. Once released into the synapse, the neurotransmitters may bind to special molecules, called "receptors" (e.g., dopamine receptor), that are located within the cell membranes of the dendrites of the adjacent neurons. This, in turn, stimulates or inhibits an electrical response in the receiving neuron's dendrites. While some neurotransmitters act as chemical messengers, carrying information from one neuron to another, other neurotransmitters (e.g., endorphin) act as neuromodulators, modulating the re-uptake of other transmitters (e.g., dopamine) back to the axon of the originated neurons. This mechanism is important for maintaining the normal activities of neurons. For example, disturbance of dopamine transmission can cause Parkinson's disease, in which a person loses the ability to execute smooth, controlled movements. Drugs like cocaine inhibit the re-uptake of dopamine, affecting the pleasure system of the brain.

For the Banff demo, a query endpoint was created from which to issue a query by identifying and locally aggregating the data sources necessary to answer the scientific question associated with our use case into a data warehouse. However, most users would prefer to ask a scientific question from a single interface, with answers from relevant sources, regardless of whether the source is remote or local, and without being required to know which data sources have information related to their question. This scenario points to the need for federating queries that can reach out to remote triplestores, relational databases, and data repositories. Data distribution is an underlying notion in the Semantic Web, where the amount of data that can be practically aggregated into a data warehouse is limited by hardware and software constraints. Furthermore, we have already mentioned the maintenance problems that stem from the data warehouse approach. In practice, resources will periodically appear on the Web that can be useful for various types of research. This makes federation attractive because it can make it possible to query data where it resides without the level of effort and infrastructure needed to add it to a data warehouse.

As illustrated in Figure 1, it is important to integrate data across different levels to support integrative neuroscience research. Such data integration presents a great challenge as different types and levels of data are available in different formats and are widely scattered over the Internet. As listed in the Neuroscience Database Gateway (NDG) [17, 18], there are nearly 200 neuroscience databases accessible over the Web. This is by no means an exhaustive list. In addition, there are web resources that are not databases (e.g., literature). This paper describes how we meet the data integration challenge by using a range of Semantic Web technologies. Instead of using a warehouse approach, we embark on a journey toward the query federation approach by developing different interfaces to meet different needs and purposes.

## Methods and results

This section first describes the data sets that we have used for query federation. These publicly available data sets represent diverse types of neuroscience and biomedical data that are provided and maintained by different sources in different formats. Some of these data sets are available in RDF/OWL format via Linked Data or SPARQL endpoints, while others are available as relational databases or files/documents in other formats such as XML, HTML, and spreadsheet. While each of these data sets serves a specific purpose, combining them provides a broader context for supporting integrative or translational research. We describe how to implement federation and description of these data sets using a range of semantic web technologies/approaches including SPARQL endpoints, standard-based middleware technologies, query translation, and voiD. The scope of our work is broad in the sense that a wide range of techniques and diverse types of data sets with heterogeneous formats are covered.

### Data sets

Table 1 gives an overview of the datasets that were used in the query federation scenario. These datasets originate from different sources and span diverse biomedical domains. Their integration can enable cross data validation, discovery of new hypotheses, and more powerful integrative analyses.

**Table 1:** List of data sources used for demonstrating query federation. Underlined entities have URIs/identifiers that are shared among the distributed datasets, thereby forming a coherent network of statements and making a federated query over all datasets possible.

Data source	Web accessible format	Typical content of statements captured in RDF/OWL (transcribed into natural language)	Used by
HCLS Knowledge Base (DERI Galway)	SPARQL query endpoint, Linked Data	*"Entrez Gene entry 2550* is the topic of the scientific journal *article with PubMed ID 9373149*. It describes a gene that encodes *senselab:GABAB_receptor* [...]"	Receptor Explorer, AIDA
HCLS Knowledge Base (FU Berlin)	SPARQL query endpoint, Linked Data, Web View, Translator	SenseLab ontology contains information about genes and neuron receptors – asking which neurons have I_K currents	AIDA, Rule Responder
DBpedia	SPARQL query endpoint, Linked Data	*"dbpedia:GABAB_receptor* is described in *Entrez Gene entry 2550*. It is a transmembrane receptor. [...]"	Receptor Explorer, FeDeRate
Mapping file between SenseLab receptors (described in HCLS Knowledge Base) and DBpedia	Static RDF/OWL document	*"senselab:GABAB_receptor* refers to the same entity as *dbpedia:GABAB_receptor* *"*	voiD, Receptor Explorer
Description of datasets and mappings (voiD file)	Static RDF/OWL document	"SenseLab ontology contains information about neuron receptor and has mapping files that link this dataset with DBpedia"	voiD
aTag collection/biological statements	Static HTML document with embedded RDF/OWL (RDFa)	"The statement 'GHB was found to bind to GABAB' in Pubmed abstract 123 was tagged with the entities *dbpedia:gamma-Hydroxybutyrate*, *dbpedia:Binding_(molecular)* and *dbpedia:GABAB_receptor* [...]"	FeDeRate
Faviki	Dynamically generated HTML with embedded RDF/OWL (RDFa)	"The web page http://example.com/ has been tagged with the entities *dbpedia:GABAB_receptor* and *dbpedia:Pharmacology* by user John_Doe [...]"	FeDeRate
IUPHAR Receptor Database	Relational database, spreadsheet, HTML	"The GABAB receptor is described in *Entrez Gene entry 2550*. It is activated by the pharmacological substance Baclofen. Gene knockout can lead to seizures. [...]"	FeDeRate

We have created two health care and life science knowledge bases (HCLS KB's) at two sites (DERI Galway and Freie Universität Berlin) using different triplestore technologies (Virtuoso and AllegroGraph), created and included data sources that are in other formats (such as spreadsheets, tags, and RDFa), and used RDF/OWL data sources created by others.

The *HCLS KB at DERI Galway* contains over 400 million RDF statements. Major constituents of the HCLS KB at DERI are the Neurocommons knowledge base [15] and the datasets generated by the "Linked Open Drug Data" task force [19] of the W3C. The Neurocommons Knowledge base consists of a sizeable collection of RDF/OWL versions of biomedical databases and ontologies, such as the Open Biomedical Ontologies (including the Gene Ontology), MEDLINE, Gene Ontology Annotation (GOA), Medical Subject Headings (MeSH), the Brain Architecture Management System, parts of the SenseLab neurobiology databases and others. The Linked Open Drug Data datasets contain pharmacologically and medically relevant datasets dealing with data about drugs, pharmacokinetcs, disease-gene associations, clinical trials data and related topics.

Data in the HCLS KB at DERI Galway are exposed in accordance to the 'Linked Data' paradigm: For example, the URI http://hcls.deri.org:8080/commons/record/ncbi_gene/2550 is used to identify the Entrez Gene record 2550 inside the knowledge base. Resolving this URI in a common web browser yields the human-readable representation of the linked data about this record that is contained in the knowledge base. If the same URI is resolved by an RDF-enabled client, the client can extract these statements in machine-readable RDF format. Entities that are referenced in statements as subjects, predicates or objects are identified by Linked Data URIs as well. This makes it possible for RDF-enabled clients to incrementally 'crawl' the web of linked data that surrounds any given database record or other entity. As an alternative to this incremental, entity-by-entity exploration, the HCLS KB at DERI can also be queried through a SPARQL endpoint http://hcls.deri.org/sparql.

The *HCLS KB at Freie Universitaet Berlin* [20] uses the AllegroGraph RDFStore as a high-performance, persistent RDF graph database. It supports SPARQL, RDFS++, and rule-based Prolog reasoning from Java applications. The knowledge base contains complementary data such as parts of the SenseLab neurobiology databases and associated traditional Chinese medicine, gene and diseases information [21]. SPARQL endpoints, Linked Open Data, and web views (using AGWebView – Web browser server for AllegroGraph) give access to the AllegroGraph triplestore data.

In addition to the HCLS KB's, our federation involves external RDF/OWL data sources developed by third parties, such as DBpedia [11] and Bio2RDF [22, 23].

Not all the knowledge used by scientists for their research is available as structured data. A lot remains only accessible as spreadsheets or HTML web pages. Social tagging tools, such as del.icio.us [24], allow scientists to annotate HTML web pages with tags for organizing their personal knowledge and sharing with their colleagues. However, without controls, tags can be obscure and error-prone and bring gaps to the linking of the knowledge generated at different time by different contributors. In our use case study, we used two social tagging tools, namely aTags [25] and Faviki [26], both of which support the use of controlled terms for tagging and achieve bringing semantics and structure to the tags.

As part of our contribution we have developed *aTags*, which offer a simple mechanism for capturing biomedical statements in RDF/OWL format and publishing them anywhere on the web. The aTag generator prototype suggests entities from DBpedia and other domain ontologies that can be used to describe the content of simple biomedical statements in an unobtrusive 'tagging' workflow. The aTag generator functionally can simply be added to any web browser with the aTag bookmarklet. After adding the bookmarklet to the browser, a user can navigate to any web page (such as an abstract of an article on PubMed), highlight a relevant statement in the text, and click the bookmarklet. The aTag generator then allows the user to add and refine semantic tags that capture the meaning of the statement in a machine-readable format, interlinking it with existing ontologies and linked data resources. The aTag generator encodes the output as XHTML+RDFa, using parts of the SIOC vocabulary [27, 28]. The resulting XHTML+RDFa can be embedded anywhere on the web such as in blogs, wikis, biomedical databases or e-mails. An exemplary XHTML page where aTags about the GABAB neuroreceptor have been collected can be found on [29]. The RDF statements contained in this XHTML page can be programmatically retrieved using RDFa services like Swignition [30] via the URL [31].

*Faviki* provides similar functionality as aTags, but is focused on classical tagging/bookmarking of entire web pages. It proposes DBpedia URIs to users so that they can annotate web pages with common, pre-defined tags. Also, the functionality of Faviki can be used in any web browser through a Faviki bookmarklet. When loading a web page, Faviki will automatically propose a set of DBpedia URIs by using the Zemanta API [32] to analyze the content of an HTML web page and to search for appropriate URIs. It can auto-complete a tagging term using the Google search API, controlling the terms used in the tagging. Bookmarks can be browsed by tag clouds or programmatically accessed using the embedded RDFa (which associate the URIs of the web pages with the DBPeida URIs tagging those pages). Again, statements about pages tagged with"GABAB receptor" (listed in [33]) can be retrieved with the Swignition service via the URL of http://srv.buzzword.org.uk/turtle/www.faviki.com/tag/GABAB_receptor.

### Query federation

Another contribution is that we have developed a number of approaches for querying and displaying RDF data from a distributed set of repositories. These approaches include the Receptor Explorer, an application for navigating receptor related information, the AIDA Toolkit, a generic set of components supporting search and annotation of semantic datasets and FeDeRate, a query federation framework. All of the investigated approaches have the following in common:

- RDF/OWL data is retrieved at runtime from multiple distributed repositories.
- Client applications are able to access this data as if it were contained in a single virtual repository.

The approaches differ along two axes:

- The application stack layer at which the virtual repository abstraction is supported: For the Receptor Explorer, this is the Enterprise Service Bus (ESB) API exposed to client applications. For AIDA web services employed by the AIDA Search client, this is the Service-Oriented Architecture (SOA) API exposed to client applications by the WSDL. FeDeRate supports this abstraction at the SPARQL query interface.
- Generality of approach: The Receptor Explorer, although built using a generic ESB framework, is focused on a single scenario involving the retrieval and display of specific receptor-related data. The AIDA Search interface, also an end-user tool, is more general-purpose and can be utilized to explore a wide range of RDF data retrieved from multiple locations. As a federated query engine, FeDeRate has the broadest range of application with the potential to be leveraged in various RDF-based applications that query a range of datasets and repositories.

#### Receptor explorer

The Receptor Explorer is a proof-of-concept application that enables users to retrieve and navigate through a collection of receptor related information stored in multiple RDF repositories (DERI HCLS KB, DBpedia [11], Bio2RDF [23] and linkedct.org [34]) and associated web sites (Wikipedia, PubMed and ClinicalTrials.gov [35]). In this use case, "receptor" serves as a unit for data federation. The genes involved in encoding a receptor are used to retrieve related publications. The retrieved publications are in turns used to find the corresponding clinical trials and to connect researchers. Therefore, the Receptor Explorer can help translational research in terms of connecting basic neuroscience research with clinical trials. In addition, it plays a role in social networking in terms of connecting researchers studying the same receptor(s).

**Figure 2:**
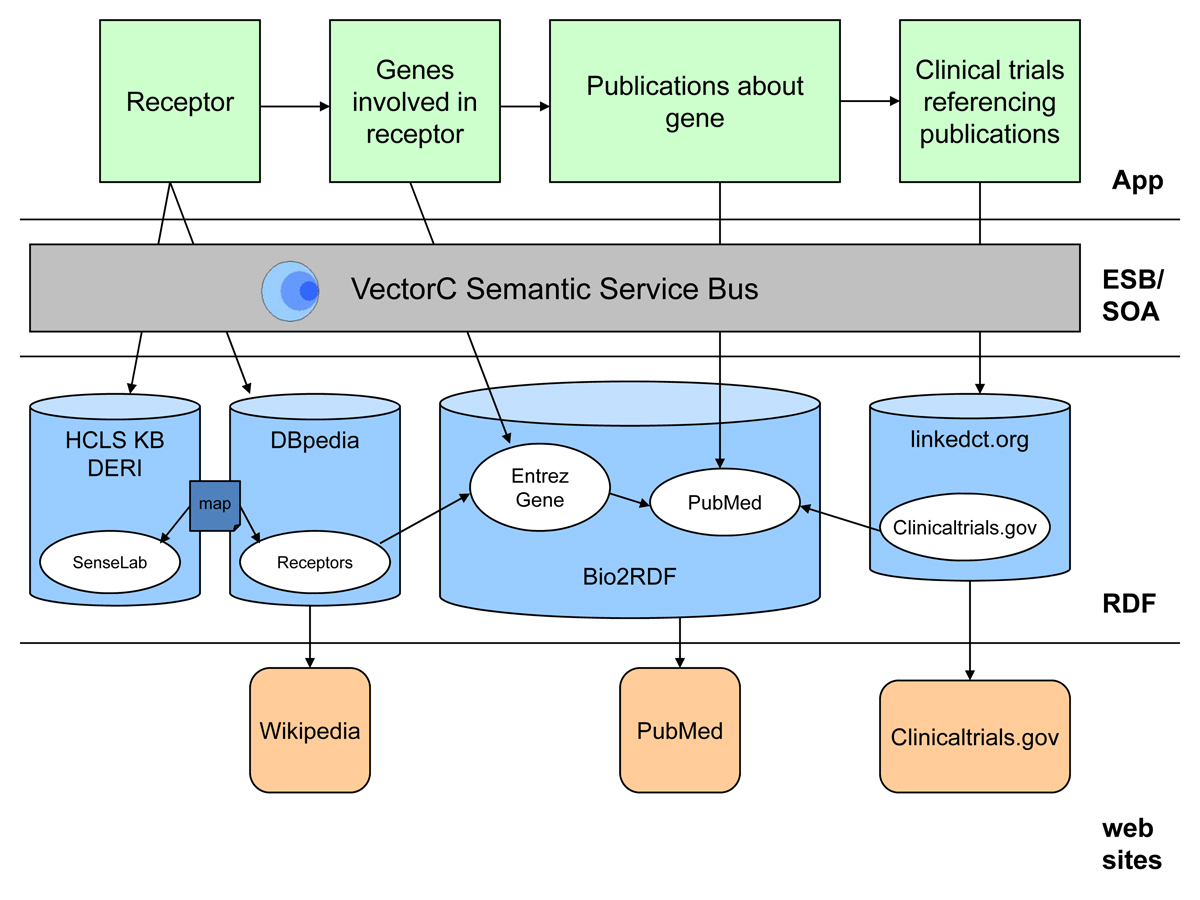
**Receptor Explorer architecture and workflow**.

As shown in Figure 2, the Receptor Explorer is comprised of a browser-based UI layered on top of a semantically enabled ESB application that communicates via SPARQL Protocol with a set of remote RDF repositories. This architecture gives the client application a federated view of the underlying RDF data by delegating client requests (e.g. retrieve the genes for a specific receptor) to ESB services that coordinate the execution of separate SPARQL queries on the various remote endpoints.

The ESB application is implemented using VectorC's Semantic Service Bus framework [36], a set of Semantic Web extensions to the Mule ESB [37] that leverages the Jena [38] and Sesame [39] frameworks. The Semantic Service Bus enables the development of applications that query, transform, route and perform reasoning over RDF data and associated ontologies with application configuration and message processing topology declaratively specified in a set of XML configuration files.

**Figure 3:**
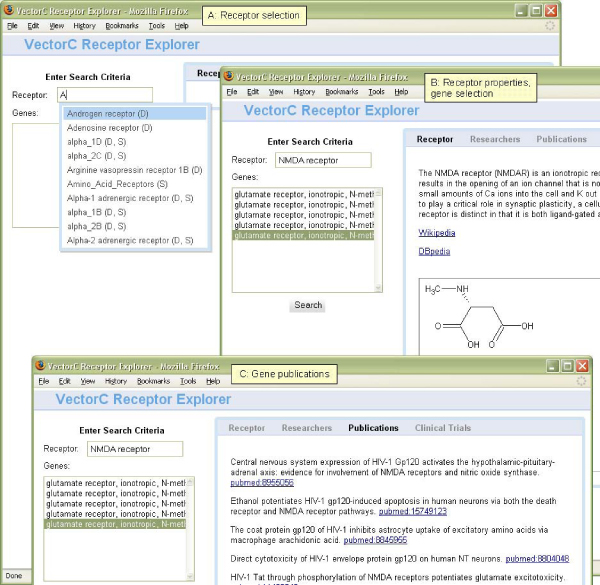
**Receptor Explorer screen shots for NMDA receptor**.

As shown in Figure 2, the Receptor Explorer queries the DERI HCLS KB, DBpedia, Bio2RDF and linkedct.org to support the following workflow:

- The user first selects a receptor from the list created by merging the DBpedia and SenseLab receptor trees (see screen shot A in Figure 3; a "D" represents a receptor from the DBpedia tree, an "S" represents a receptor from the SenseLab tree).

- The DBpedia receptor tree is rooted at http://dbpedia.org/class/yago/Receptor105608868
- The SenseLab receptor tree is rooted at http://purl.org/ycmi/senselab/neuron_ontology.owl#Neuron_Receptor
- Overlaps are computed using the SenseLab-to-DBpedia OWL mapping file [40]
- The set of genes (as Entrez Gene ids) involved in the selected receptor are retrieved from DBpedia (see screen shot B in Figure 3).
- The description and image for the receptor, if available, are retrieved from DBpedia and displayed along with hyperlinks to the associated Wikipedia and DBpedia URLs for the receptor (see screen shot B in Figure 3).
- The user selects one of the displayed genes and the set of PubMed publications that reference the gene are retrieved from Bio2RDF. The publications display includes hyperlinks to the associated PubMed URLs (see screen shot C in Figure 3).

The set of clinical trials that reference the retrieved publications are retrieved from linkedct.org. The clinical trials display includes hyperlinks to the associated ClinicalTrials.gov URLs.

#### AIDA toolkit for browsing and searching

The AIDA Toolkit [41] is directed at groups of knowledge workers that cooperatively search, annotate, interpret, and enrich large collections of heterogeneous documents from diverse locations. It is a generic set of components that can perform a variety of tasks such as learn new pattern recognition models, perform specialized search on resource collections, and store knowledge in a repository. W3C standards are used to make data accessible and manageable with Semantic Web technologies such as OWL, RDF(S), and SKOS. AIDA is also based on Lucene and Sesame. Most components are available as Web Services and are open source under an Apache license. AIDA is composed of three main modules: Storage, Learning, and Search. The modules employed in the BioRDF pilot are Search and Storage. The use case here is broader than that of the Receptor Explorer described previously. In addition to receptors, the user can query diverse types of biomedical entities such as disease, gene, pathway, and protein across different repositories including the literature.

**Figure 4:**
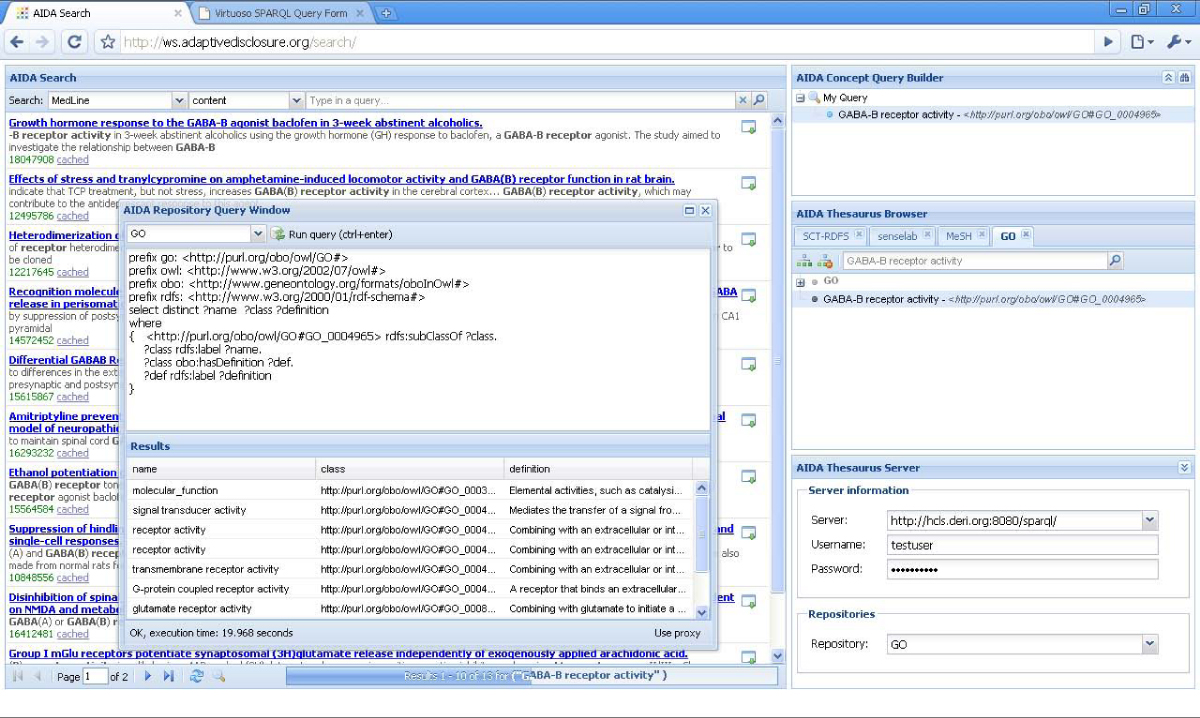
**The web interface of the AIDA Toolkit showing both the search of a Medline index and a SPARQL query of the HCLS KB at DERI using the same term**.

The web browser-based AIDA Search interface was originally designed to allow the browsing and search of vocabularies available in the SKOS (Simple Knowledge Organization System) format from Sesame repositories. Selected concepts from a given vocabulary can be employed in the search of a Lucene index available from the AIDA Search interface. Such search makes use of both the knowledge resources stored in a triplestore and a Lucene index of document resources, effectively federating triplestores and indexes. Lucene search is typically demonstrated with the MedLine index. The background of Figure 4 shows the search of the MedLine index using the GO term "GABA-B receptor activity". In order to prototype an interface for exploring knowledge base contents in BioRDF, we employed an adaptation of the AIDA Search interface that we call a 'SKOS Lense' in order to browse the subsumption hierarchies of ontologies contained in the HCLS KB. Once our Storage component had been augmented to connect to Virtuoso (outfitted with a Sesame adapter), we were able to view the class hierarchies contained in a given named graph of the HCLS KB and search rdfs:labels for strings. This made the AIDA Search interface useful for browsing and searching vocabularies, and ontologies, as well as text corpora. This is the first step in a prototype application designed to support query building and federated query.

An example query scenario where the 'SKOS Lense' would be useful is in the case that the user wants to query about a particular term but does not know the valid identifier to use for that term in the query (some demo queries with their English translations can be found on the DERI web page [42]). It is possible to use the AIDA Search interface to search through the actual rdfs:labels of the named graph for the Gene Ontology (GO) in the DERI HCLS KB for "GABA-B receptor activity" and drag the desired term to the Query Builder. The term that is added to the (SPARQL) query is the corresponding GO id (with namespace) that is useful in the HCLS KB SPARQL query (see Figure 4). In such a way, we have made it possible for the user to work with a human-readable label "GABA-B receptor activity" instead of a machine-based id "http://purl.org/obo/owl/GO#GO_0004965", yet build a valid SPARQL query. The same web interface can be used to browse and search a medical vocabulary such as SNOMED-CT or MeSH from, for example, a Sesame HCLS Terminology repository hosted at Duke University in order to build and execute a query on the Virtuoso HCLS KB at DERI or Allegrograph HCLS KB at the Freie Universität Berlin.

#### FeDeRate

Working at a lower level than AIDA or Receptor Explorer, FeDeRate offers a step in the direction of enabling the system to integrate resources without procedural programming or warehousing. This tool uses a simple, practical approach to federate queries over multiple remote data stores, including relational databases, XML and XHTML documents, and Semantic Web data stores. The approach uses a SPARQL query (called the "orchestrating query") to specify a series of graph patterns to retrieve from remote SPARQL services. The order of the graph patterns provides a query orchestration, and variables shared between the graph patterns express the way information from these remote sources is joined. The use case of FeDeRate is that higher level tools like Receptor Explorer and AIDA can use FeDeRate to implement query federation not only against triplestores but also relational stores. Below we provide a technical discussion of FeDeRate.

The following query gathers receptor data from **source1** and **source2**, connecting them by a common human EntrezGene identifier:

SELECT ?iupharNm ?type ?label

...

GRAPH < source1 > {

?iuphar   iface:iupharName   ?iupharNm .

?human   iface:iuphar   ?iuphar .

?human   iface:geneName   "GABBR1" .

?human   iface:entrezGene   ?humanEntrez }

GRAPH < source2 > {

?gene   db:entrezgene   ?humanEntrez ;

?gene   a   ?type ;

?gene   rdfs:label   ?label }

A researcher would compose such a query by forming a question, considering the popular expression of the data that question touches, and locating the resources serving this data. The ease of re-use of ontologies in the Semantic Web and the existence of a standard protocol for the SPARQL query language have led to a proliferation of resources serving queries like this. (Consider the alternative in relational databases where database administrators immerse themselves in proprietary software to create data warehouses, or provide non-standard language extensions sitting above SQL.)

FeDeRate determines which variables in each GRAPH constraint are present in the SELECT, or are referenced in subsequent graph patterns. Starting with the first GRAPH constraint, FeDeRate translates the constraint into a subordinate SPARQL query.

SELECT ?iupharNm ?humanEntrez

FROM < source1 >

WHERE {

?iuphar   iface:iupharName   ?iupharNm .

?human   iface:iuphar   ?iuphar .

?human   iface:geneName   "GABBR1" .

?human   iface:entrezGene   ?humanEntrez }

Subsequent subordinate queries are accompanied by bindings constraining the variables bound by earlier queries. These can be expressed either as standard SPARQL constraints:

FROM < source2 > WHERE {

?gene   db:entrezgene   ?humanEntrez ;

?gene   a   ?type ;

?gene   rdfs:label   ?label

FILTER (?humanEntrez = 2550 ||

?humanEntrez = 9568)

}

or by an extension to SPARQL called SPARQLfed, which ships bindings around in a tabular form:

... ?gene   rdfs:label   ?label }

BINDINGS (?humanEntrez ?var2 ...) {

(2550   <val1> ...)

(9568   <val2> ...)

}

SPARQL is an effective language for unifying data over diverse sources because it has a simple data model (triples of subject, relationship, object) which can express existing data formats, e.g. relational databases or spreadsheets. We pay particular attention to the relational database, as it holds the majority of the computer-processed data in the life science domain. It is common that each record in a database describes some object in the domain of discourse. For example, a biological pathway database will have records for different pathways and records for the genes involved in those pathways. In this respect, each column in the record asserts some property about the object of discourse, an assertion which is written in RDF:

   @prefix db: <http://pathway.example/Pathways>.

db:APOE db:is_involved_in db:Alzheimer_disease_pathway.

The prefix "db:" is associated with a unique identifier for this database. Given a unique identifier, it is easy to imagine the RDF view of that database. It was asserted earlier that RDF encourages the sharing of terms, but the terms in our (unmaterialized) RDF view of the database are specifically unique. There are many different schemes for mapping relational data to RDF; FeDeRate uses a SPARQL CONSTRUCT to map from the rather proprietary view of the database described above to terms chosen to be shared with others in a community. The community can range from world-wide to intra-laboratory; a sharable data format benefits anyone who will share any of their data with anyone else.

We used FeDeRate to connect some of the data sources described in Table 1. We started with a relational database containing data from the IUPHAR database of receptor nomenclature and drug classification [43, 44]. This database includes entries that contain the Entrez Gene identifier 2550 (which refers to the gene encoding the GABAB receptor). A SPARQL CONSTRUCT mapped this very flat database to a more structured RDF form (excerpt below):

CONSTRUCT {

?iuphar   interface:family   ?family .

?iuphar   interface:iupharName   ?iupharName .

?human   interface:iuphar   ?iuphar .

?human   interface:entrezGene   ?human_Entrez_Gene.

} WHERE {

?a   receptors:Family   ?family .

?a   receptors:Official_IUPHAR_name   ?iupharName .

?a   receptors:Human_Entrez_Gene   ?human_Entrez_Gene .

}

An identifier associated with the "receptors:" prefix provides the proprietary RDF view of the receptors database while terms with the "interface:" prefix would be a product of e.g. community consensus amongst neuroscientists. The mapping (above) creates another view of the database allowing users to ask questions in terms of the shared ontology. Neither the proprietary nor interface views were ever materialized. The subordinate queries, expressed in terms of the interface data structure, were passed to a SPARQL server which was configured with the database identifier and the CONSTRUCT mapping. This server used the CONSTRUCT mapping to calculate a query which would work over the proprietary view, and then used the databases identifier to transform that query into a SQL query. As a result, that portion of the query was executed directly on the relational database in a single SQL query.

Our orchestration queries went on to join the IUPHAR data with data from aTags and DBpedia. Examples include asking for all the properties of a particular receptor, which would supplement the information that a Linked Data browser could present to a user, and asking for specific properties, like the supertypes of a particular protein.

While we used FeDeRate's transformation rules to map between proprietary and interface data structures, they can be used for mapping between arbitrary schemas, with the same execution efficiency. The interface side of CONSTRUCT mappings constitute descriptions of the data available at query services. These descriptions can be passed to users and researchers, helping them compose orchestration queries. The interface patterns may also be used by tools which associate these patterns with services in order to automatically generate orchestration queries, allowing users to ask questions without knowing which services are needed to answer them. Methods like voiD can complement these descriptions, providing the necessary information for network query optimizers which consume SPARQL queries and equivalent queries, orchestrating efficient queries over sets of services.

### Description of linked data sets

To show how query federation may benefit from the machine-readable description of data sets, we created the mapping of NeuronDB receptor and neuron classes, (part of the SenseLab ontology) with DBpedia resources. The mappings were expressed using owl:sameAs statements. Part of the mapping was automatically created using the trial version of TopBraid Composer 3 beta [45], a commercial ontology editor and Semantic Web platform. The automatic mapping result was then manually corrected.

In order to help application developers to understand how best to query these datasets and to use the mapping information, we adopted the voiD to describe the two datasets and their mappings. By the time of writing, this is the first real example demonstrating how voiD can be used to describe research datasets.

The great number of openly accessible datasets makes the integration of life science data more viable. However, the challenge remains to automate the discovery and selection of appropriate datasets needed by an application, and to understand how best to query the data and optimize these queries. Linked data method encourages data providers to publish links between their datasets and others. Being able to automatically locate such information will provide a higher point of departure for data integration. VoiD is proposed to enable the discovery and usage of linked datasets or datasets accessible through SPARQL.

VoiD is a RDFS-based vocabulary [46] and a set of best practice instructions [47] guiding the publication of and extension to voiD. The principle of the voiD effort is to use real requirements to guide the scope of the design, and to re-use existing vocabulary wherever possible. Therefore, the creation of new classes and properties under the voiD namespace http://rdfs.org/ns/void# is kept to the minimum. Currently, voiD defines two classes in the voiD namespace: a *void:Dataset* to represent a set of RDF triples that are published, maintained or aggregated by a single provider, and a *void:Linkset* to represent the interlinking between datasets. Details about the vocabulary can be found at [48].

**Figure 5:**
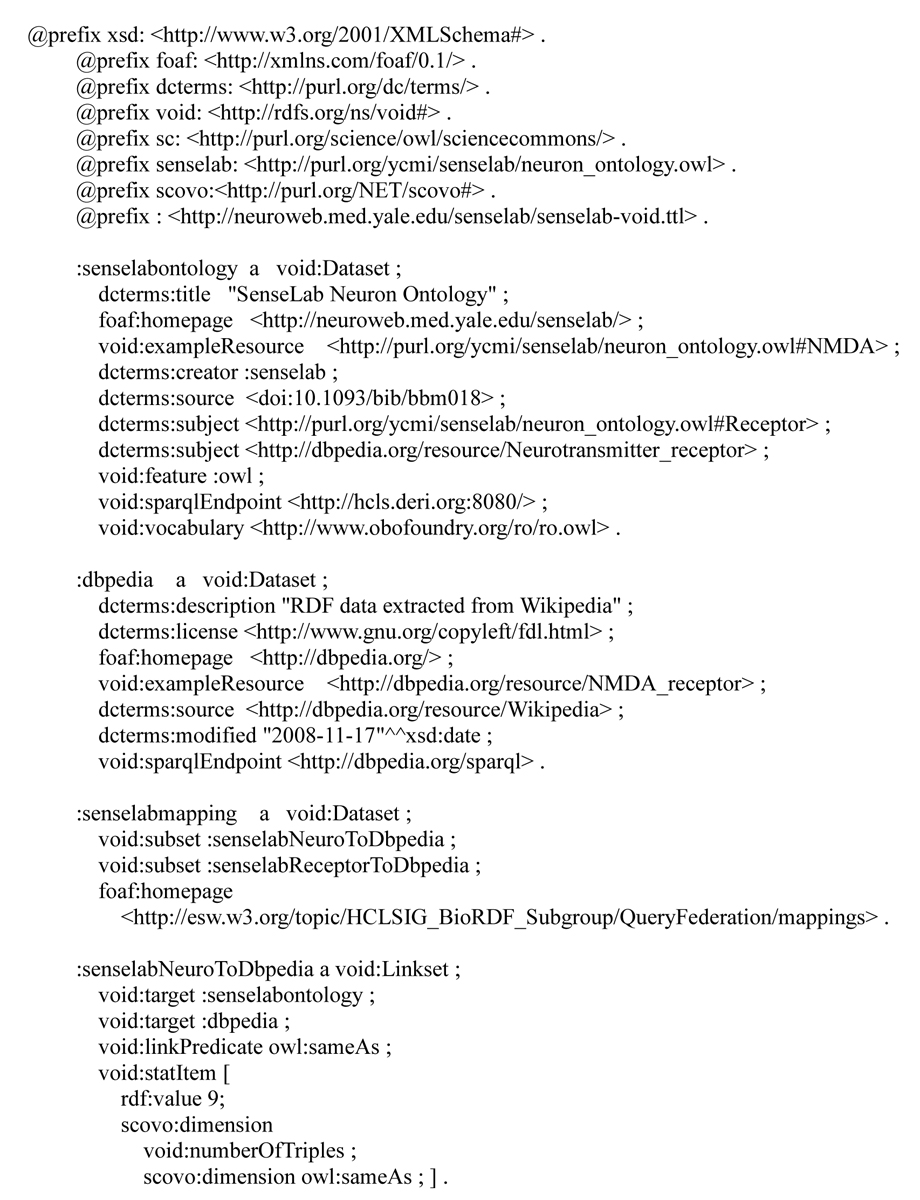
**A sample voiD description describing the SenseLab and DBpedia datasets and the mapping between these two datasets**.

Figure 5 shows a sample voiD description of the SenseLab and DBpedia datasets and the mapping between these two datasets. It provides information about the following:

- The SenseLab dataset (*:senselabontology*) is published by Senselab (*:senselab*) and contains information about receptors and neurotransmitter receptors. This information about the dataset can be used by application developers to discovery datasets relating to neuro receptors. Application developers can also use the SPARQL endpoint and example URIs given in the voiD description to start queries to this dataset.
- The DBpedia dataset (*:dbpedia*) used in our use case is openly accessible under the GNU license and last updated on "2008-11-17".
- The mapping between *:senselabontology* and *:dbpedia* is published as a separate dataset, i.e., *:senselabmapping*, which is available at http://purl.org/ycmi/res/senselab_receptor_to_DBpedia_mapping.owl. The property void:subset defines that, conceptually, there are two sets of links published in this mapping file, one mapping the neuro classes (*:senselabNeuroToDbpedia*) in the two datasets and the other mapping the receptor classes (whose voiD descriptions are omitted here).
- *:senselabNeuroToDbpedia* is one set of the mappings. Application developers can use the voiD description about this void:Linkset to understand the type of the links created for Senselab and DBpedia, to find out the number of links created in the mapping, etc. We could also publish example neuro URIs to guide the query federation of these data sources.
- The complete voiD description can be found at [49] VoiD descriptions can be written in any RDF format (such as RDF/XML and Turtle). One recommended way to publish a voiD description is to advertise it in the Semantic Sitemap [50], which is an extension to conventional sitemap files for describing the datasets hosted by a server. In this way, the voiD descriptions can be consumed by semantic search engines like Sindice [51, 52] or data query federation engines like FeDeRate to provide dynamic federation of distributed datasets.

## Discussion

Triplestore technologies have helped speed SPARQL query performance and make management of large numbers of triples efficient. The different triplestore technologies, however, make it impossible to have cross-access to the datasets residing in separate triplestores. SPARQL can help solve this issue of interoperability by allowing datasets in each triplestore to be accessed via standard SPARQL queries issued by clients (or by middleware) to the common SPARQL endpoint service. This approach allows cross-links to be created at the programming level. Linked Data is a different way of publishing data on the Semantic Web that makes it easy to interlink, discover, and consume data on the Semantic Web. Linked data browsers (e.g., Tabulator [53, 54]) have been developed to allow the user to display data and traverse the links. In addition, it allows semantic web crawlers to crawl the linked datasets for indexing purposes. Recently, third party tools such as Pubby [55] have become available for creating linked data interface to SPARQL endpoints. In addition, triplestores like Virtuoso provide the linked data option as part of their functionality. In addition to making links and supporting data browsing, Linked Data should support flexible querying. Projects like SQUIN [56] have begun to address this.

**Figure 6:**
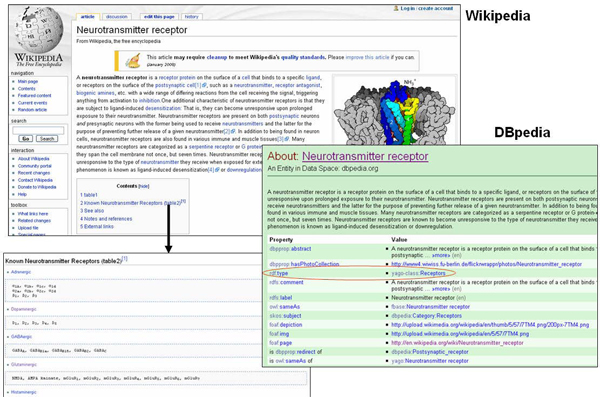
**Semantic mismatch between Wikipedia and DBpedia for neurotransmitter receptors**.

While we were in the process of establishing receptor mappings between SenseLab and DBpedia, we discovered a semantic mismatch between Wikipedia and DBpedia in terms of neurotransmitter receptor classification. As shown in Figure 6, there is a Wikipedia article page describing neurotransmitter receptors (receptors that bind to neurotransmitters, http://en.wikipedia.org/wiki/Neurotransmitter_receptor). On that page, a table is provided that lists the known neurotransmitter receptors. As shown in the table, different types (classes) and subtypes (subclasses) of neurotransmitter receptors have been identified. For example, glutaminergic receptor is a type of neurotransmitter receptor and NMDA receptor is a type of glutaminergic receptor. When we compared this information with the structured description of neurotransmitter receptors in DBpedia http://dbpedia.org/page/Neurotransmitter_receptor, we noticed that neurotransmitter receptor is defined as an instance of the class "receptors" in DBpedia (circled in red). In other words, neurotransmitter receptor cannot have any subclasses, which is not consistent with its definition in Wikipedia. We reported such an inconsistency to DBpedia and the problem will be addressed. One outcome of our work is that it has led to an improved quality of DBpedia. In the future, these manual corrections can also be complemented by automated procedures, such as described in [57].

The need for common URI identifiers is the following: in order to perform data integration or machine inference across multiple data repositories, we must be able to unify on the elements of the triples that we are using. Another requirement is that we do not want the truth values for statements based on these identifiers to change because the identifiers change. For example, if the object of one triple is the name of a protein (e.g. APOE) and the object of another triple has precisely the same protein with the same name, then we can connect the triples and, effectively connect the RDF graphs in which they are stored. Such unification is hard unless exactly the same identifiers have been used to refer to the same things, i.e. unless the identifiers are semantically-equivalent URI's. Unfortunately, due to the unavailability of a central registry, the tendency on the Web is towards both synonymous identifiers, i.e. different identifiers that refer to the same thing (e.g., different URI's have been used to identify "dopamine receptor" by different sources such as DBpedia, SenseLab and BioPortal) and polysemy, or the use of the same name to refer to different things (e.g. 'spine' to refer to 'dendritic spine' or 'spinal cord'). These problems can be verified by visiting the NCBO BioPortal [58] and searching for a particular protein or disease. Many essential concepts are reused in different ontologies and thereby acquire different URI's. If the semantics of the unmatched identifiers have been formalized, ontology alignment could be required in order to match them. If the semantics of the synonymous URI's haven't been formalized, the process of alignment can be even more costly and uncertain, sometimes requiring an interview process with the responsible parties. Therefore, it is much more robust if the semantics of the identifier is available as RDF from the dereferenced HTTP URI. The use of human-readable names as identifiers has a few disadvantages such as the fact that names can be deceiving and names can change. Consider the name of the disease *mycosis fungoides*. The name actually refers to a non-Hodgkins lymphoma, a disorder that has nothing to do with fungus but whose name implies a fungal relation due to a historical artifact. If we had classified this disease as a fungus-related disorder because of its name, this could have led us to faulty conclusions. In a second example, consider two data sources that refer to the dopamine receptor, one as 'dopaminergic receptor', the other as 'dopamine receptor'. Such data sources would not unify if they relied on human-readable identifiers even though they refer to exactly the same thing. A central registry that can supply stable access to permanent identifiers could alleviate these problems. One attempt to establish a reliable and stable system for such identifiers is the Shared Names initiative [59]. In addition, scientific domain authorities play an important role in URI harmonization and nomenclature standardization.

Workflow bears some similarity with query federation in the sense that it orchestrates services including query services to perform a (complex) task. The results or data produced by a workflow can be fed as input to other workflows. Data provenance (tracking how immediate data sets are generated) is an important topic in workflow. It is also relevant to query federation. For example, the integrated data/results produced by a particular query federation task can be tracked using a data provenance approach.

As rules are part of the Semantic Web stack (see [60, 61]), query federation can benefit from the use of rules and rule chaining. For example, a rule may be devised to infer a certain type of receptors as potential drug targets if such receptors are found highly expressed in a brain region under a certain neurological disease state as compared to the normal state. Semantic Web Rule Language (SWRL) [62] and Rule Interchange Format (RIF) [63] are two broad efforts of introducing rules to the Semantic Web (see [64] for an overview). The Rule Responder system [65] uses distributed rule-based inference services which are deployed on an enterprise service bus to implement complex conditional decision logic, distributed delegations/process logic as well as dynamic reaction logic. The Rule Responder middleware can directly be built on top of FeDeRate to access data via federated SPARQL queries and process this data using the rule-based inference services to answer and prove complex conditional research queries.

## Conclusion

We have demonstrated the use of a set of state-of-the-art Semantic Web technologies in support of a neuroscience query federation scenario. The federation approaches presented in our paper meet different purposes. The AIDA approach is intended for someone who wants to explore the options by browsing and/or querying broadly against different data sources. It supports different search interfaces including text-based searching and SPARQL querying. The receptor explorer is designed for users who have a very specific focus. In this case, it allows receptor-based queries to be performed across different data sources. The user interface is designed for someone who may not be familiar with SPARQL. Finally, FeDeRate is a query translation approach that is used by developers for implementing software systems that support query federation across multiple data sources in different formats, including legacy data sources such as relational databases.

While named graphs allow identification of data graphs, voiD can be used to provide a structured summary of the types of data contained in a single graph or interlinked graphs. Our exercise of describing datasets using voiD has demonstrated the feasibility of this vocabulary for capturing key information about datasets that can be used to facilitate data discovery, such as the subject of the dataset, and to facilitate the use of the dataset, such as the SPARQL endpoint of the dataset. For example, a dataset containing a list of receptors may be summarized to have the data type "receptor", and a different dataset containing a list of drugs may be summarized to have the data type "drug". These voiD descriptions then could be used to reveal these datasets to a data link creator who is interested in establishing links between drugs and receptors (e.g., some receptors are targets of certain drugs). VoiD can also be published for each dereferenceable URI of a dataset to define, for example, the dataset that this URI belongs to, allowing one to "follow-the-nose" [66].

While the Semantic Web offers a global data model including the use of Uniform Resource Identifiers (URI's), the proliferation of semantically-equivalent URI's hinders large scale data integration. In addition, the quality of data in terms of both content and structure has a great impact on the value of query federation. Our work helps push the envelope of these Semantic Web technologies in relation to the biosciences. It also helps stimulate further research, investigation, and participation at the community level. Our journey will lead us to a new frontier of life science web that enables federation of machine-readable and human-readable web resources.

### Future directions

We have identified the following areas for guiding our future work.

- We will identify new use cases including new data sets, which will demonstrate data integration of basic, clinical and translational neuroscience (or biomedical) research. We will incorporate new datasets as deemed appropriate into HCLS KB's to increase both the scope and power of query federation.
- We will work with different communities including ontology and neuroscience communities to address areas such as URI harmonization, which require both technical and social collaboration.
- We will explore how to expand the individual federation approaches described in the paper as well as investigate how to combine them to increase the power of query federation. For example, with the maturation of voiD and its wider adoption by linked data publishers, we are expecting more full-fledged, dynamic data query federation enabled by the voiD descriptions about distributed data sources on the Web. For example, we may restrict queries to datasets based on voiD descriptions such as date and size. A combination between voiD query engines and a rule-based query federation approach [65] promises a powerful federation system in support of translational research.
- We will work on creating web applications that make creating, publishing and searching of RDF/OWL intuitive and simple for biomedical researchers and clinicians.

## Availability and requirements

AIDA Toolkit: The software is available at http://www.adaptivedisclosure.org/aida and may be freely downloaded under the Apache license.

FeDeRate: The data and software are available at https://sourceforge.net/projects/swobjects and may be freely downloaded and used with no license requirements.

HCLS KB hosted by DERI, Galway, Ireland http://hcls.deri.org/sparql

HCLS KB hosted by the Freie Universitaet Berlin: http://www.corporate-semantic-web.de/hcls.html
